# A cuproptosis score model and prognostic score model can evaluate clinical characteristics and immune microenvironment in NSCLC

**DOI:** 10.1186/s12935-024-03267-8

**Published:** 2024-02-10

**Authors:** Yijie Tang, Tianyi Wang, Qixuan Li, Jiahai Shi

**Affiliations:** 1 Nantong Key Laboratory of Translational Medicine in Cardiothoracic Diseases and Research Institution of Translational Medicine in Cardiothoracic Diseases, Affiliated Hospital of Nantong University, Nantong University, Nantong, 226001 Jiangsu China; 2 Department of Thoracic Surgery, Affiliated Hospital of Nantong University, Nantong, 226001 Jiangsu China; 3 School of Public Health, Nantong University, Nantong, 226019 Jiangsu China

**Keywords:** Cell death, Copper, Gene expression profiling, Prognosis, cluster analysis, Carcinoma, non-small-cell Lung Cancer

## Abstract

**Background:**

Cuproptosis-related genes (CRGs) are associated with lung adenocarcinoma. However, the links between CRGs and non-small-cell lung cancer (NSCLC) are not clear. In this study, we aimed to develop two cuproptosis models and investigate their correlation with NSCLC in terms of clinical features and tumor microenvironment.

**Methods:**

CRG expression profiles and clinical data from NSCLC and normal tissues was obtained from GEO (GSE42127) and TCGA datasets. Molecular clusters were classified into three patterns based on CRGs and cuproptosis cluster-related specific differentially expressed genes (CRDEGs). Then, two clinical models were established. First, a prognostic score model based on CRDEGs was established using univariate/multivariate Cox analysis. Then, through principal component analysis, a cuproptosis score model was established based on prognosis-related genes acquired via univariate analysis of CRDEGs. NSCLC patients were divided into high/low risk groups.

**Results:**

Eighteen CRGs were acquired, all upregulated in tumor tissues, 15 of which significantly (*P* < 0.05). Among the three CRG clusters, cluster B had the best prognosis. In the CRDEG clusters, cluster C had the best survival. In the prognostic score model, the high-risk group had worse prognosis, higher tumor mutation load, and lower immune infiltration while in the cuproptosis score model, a high score represented better survival, lower tumor mutation load, and high-level immune infiltration.

**Conclusions:**

The cuproptosis score model and prognostic score model may be associated with NSCLC prognosis and immune microenvironment. These novel findings on the progression and immune landscape of NSCLC may facilitate the provision of more personalized immunotherapy interventions for NSCLC patients.

**Supplementary Information:**

The online version contains supplementary material available at 10.1186/s12935-024-03267-8.

## Introduction

Lung cancer, which has the highest morbidity and mortality in the world [1], can be pathologically classified into non-small-cell lung cancer (NSCLC) and small-cell lung cancer (SCLC). NSCLC accounts for almost 85% of all lung cancer cases according to the global tumor statistics [2] and leads to nearly 1.35 million deaths worldwide each year [3]. The treatment of NSCLC involves surgery, radiation therapy, chemotherapy, and targeted drug therapy [4]. Immunotherapy, which involves the use of inhibitors such as nivolumab and gefitinib, has recently replaced traditional platinum-based chemotherapy [4]. These inhibitors are antibodies that target check-points such as programmed cell death-1 (PD-1) to prevent immune suppression [5] and epidermal growth factor receptor (EGFR) to regulate cell growth, proliferation, and differentiation [6]. However, the relative survival rate in advanced NSCLC is still approximately 15% [7]. Despite computed tomography (CT) has reduced the mortality rate of early NSCLC by 20% [8], and the application of PET-CT has greatly improved the precision of NSCLC grading, merely depending on imaging methods alone are still unable to more accurately assess the development or prognosis of NSCLC patients and choice of treatment strategy [9, 10]. Hence, establishing a viable biomarkers and prognostic models, thereby revealing the prognostic genetic features of NSCLC in clinical practice is of considerable significance.

Cell death pathways, such as apoptosis, pyroptosis, and necrosis, are critically important in all living tissues and are essential in cancer prevention [11, 12]. Cell death pathway inhibition seems to be involved in cancer development and progression [13]. Ferroptosis, an iron-dependent cell death pathway is a of interest [14]. Ferroptosis promotes cell death by glutathione level depletion, glutathione peroxidase (GPX4) activity decrease, suppression of the metabolism of lipid oxides, and reactive oxygen species production [15]. Understanding the importance of ferroptosis in cancer has prompted investigation of other cell death pathways. Similar to iron, copper is also an indispensable element in the human body, albeit in minor quantities. Recently, the term “cuproptosis” was suggested to describe copper ion-dependent cell death, when Cu2 + accumulates excessively in mitochondrial respiration-dependent cells [16]. The mechanism is thought to involve an increase in insoluble dihydrolipoamide acetyltransferase (DLAT), which is part of the pyruvate dehydrogenase complex, leading to cytotoxicity and cell death [16]. Copper ion metabolism disorder has been associated with tumor growth and metastasis, collagen remodeling, and immune response in various cancers [17–19]. Correlations have been found between cuproptosis-related genes (CRGs) or long non-coding RNAs (lncRNAs) and the development of a number of cancer types, including breast cancer [20], melanoma [21], lung adenocarcinoma [22], and bladder cancer [23]. Previous studies revealed that long non-coding RNA signature may be related to cuproptosis and may predict clinical outcomes of LUAD [24–26] and recent studies also have presented the similar cuproptosis-related signature to predict prognosis and immune infiltration in lung adenocarcinoma [27, 28], however, our research established two signatures including cuproptosis score model and prognostic score model to evaluate the development and prognosis not only in lung adenocarcinoma but also in lung squamous carcinoma, meanwhile, we have also analyzed the correlation of clinical features, tumor microenvironment and potential therapy based on the two models: cuproptosis score model and prognostic score model, which previous research has never been conducted before, we hope our study will provide another approach to percept into clinical features and the mechanism of cancer progression in NSCLC.

Therefore, the aim of this study was to investigate CRDEGs in NSCLC. Based on the Gene Expression Omnibus (GEO) and the Cancer Genome Atlas (TCGA) datasets we first investigated the expression of CRGs in 1282 NSCLC samples. Then, using 1829 intersection genes from CRG subtype patterns we constructed CRDEG prognostic score models through a series of bioinformatics methods, including prognosis analysis and tumor microenvironment (TME) feature analysis. Prognosis-related genes were acquired by univariate analysis, and a cuproptosis score model was established and coupled with clinicopathological and immune-related analysis similar to the one performed in the previous model. In this way we aimed to explore the differences between the clinical and immune characteristics of the two models, the cuproptosis and the prognostic score models.

## Methods

### Cell culture and tissue acquisition

The human lung adenocarcinoma (LUAD) cell lines A549, HCC827, and H23 were purchased from Shanghai Cell Bank of the Chinese Academy of Medical Sciences. The normal human pulmonary epithelial cell line BEAS-2B, and the LUAD cell lines H1975, H1650, and PC9 were purchased from The American Type Culture Collection (ATCC, Rockville, MD, USA). All cells were cultured in RPMI-1640 (11,875,093, Gibco, USA), 10% fetal bovine serum (10,099,141 C, Gibco, USA), 0.1 mg/mL streptomycin (10,378,016, Gibco, USA), and 100 U/mL penicillin (10,378,016, Gibco, USA) and maintained at 37 ℃ in a 5% CO_2_ atmosphere. 50 pairs of clinically NSCLC samples were obtained in accordance with the Declaration of Helsinki (2000) of the World Medical Association and study was approved by the Ethics Committee of the Affiliated Hospital of Nantong University (Nantong, Jiangsu, China) under no. 2022-L165.

### RNA extraction and quantitative real-time PCR (qRT-PCR)

Total RNA was harvested from both cells and tissues with Trizol reagent (10,296,028, Thermo, USA). cDNA was then produced using the RT Reagent kit (18,091,200, Thermo, USA) according to the manufacturer’s protocol. The mRNA expression level was estimated by a ChamQ Universal SYBR qPCR Master Mix (Vazyme Biotech Co., Ltd). Glyceraldehyde-3-phosphate dehydrogenase (GADPH) was taken as internal reference. The sequences of the primers we utilized were as follows, presented also in Supplementary Table 1 (18 CRGs):

GAPDH forward: 5’-GTCTCCTCTGACTTCAACAGCG-3’.

GAPDH reverse: 5’-ACCACCCTGTTGCTGTAGCCAA-3’.

The following qRT-PCR conditions were implemented: 95 ℃ for 5 min and then 40 cycles of 95 ℃ for 15 s, 60 ℃ for 30 s, and 72 ℃ for 30 s. The assay was performed in triplicate and the comparative quantification cycle (Cq) method (2 − ΔΔCq) was employed to estimate the relative expression level of the 18 CRGs.

### Acquisition of data source and preconditioning

The gene expression quantification profile, somatic mutant profile, copy number variation (CNV) files, and clinical files of NSCLC cases were obtained from GEO (GSE42127, https://www.ncbi.nlm.nih.gov/geo/query/acc.cgi?acc=GSE42127) and TCGA (https://portal.gdc.cancer.gov/) datasets. Using TCGA, we selected gene expression qualifications in transcriptome profiling data of lung adenocarcinoma and lung squamous cell carcinoma, excluding miRNA expression data. Then, in GEO, the original NSCLC expression data were downloaded from the platform as a GPL file. The TCGA and GEO data were integrated during data correction processing. Data of 1106 samples acquired from TCGA was post-processed, normalized, and transformed from FPKM format to TPM format by ‘limma’ package and R (4.2.1) to combine data. After removing samples which lacked clinical data or had survival data that with values less than or equal to zero, 1282 samples were obtained and subjected to analysis: 1106 from TCGA dataset (103 normal tissues and 1003 tumor tissues) and 176 tumor tissues from the GEO database. All 18 cuproptosis-relatled genes were acquired via past research and documents, which have been studied and verified to be highly correlative with mechanism or pathway of cuproptosis.

### Variance analysis between normal/tumor tissues

The differences in the expression of CRGs between normal/tumor tissues from the TCGA dataset were estimated by ‘limma’ package and R software. The CRG expression quantification profile was converted into ggplot2 input file and the Wilcoxon signed-rank test was applied to compare the expression levels in tumor and normal tissues. Five levels of significance were used with *P*-values of 0, 0.001, 0.01, 0.05, 1.

### CNV frequency and mutation type analysis of CRGs

Somatic CNV and mutation data was acquired from the TCGA and GEO datasets. CRGs and cuproptosis high/low risk CNV matrix and gene mutant matrix were sorted by perl software, ‘Rcircos’, and ‘Maftools’ packages in R software to calculate variants difference, tumor mutation load (TMB) and non-synonymous somatic mutations. Packages ‘igraph’ was adopted to realize the CNV and mutant frequency picture.

### Consensus Cluster analysis of CRGs and CRDEGs

The ‘Consensus Cluster Plus 1.60.0’ package was employed in R software to classify the CRDEGs. This is an algorithm that can be used to identify the members and number of clusters in a data set. Consistent clustering can determine the optimal number of clusters K. Consistent clustering verifies clustering rationality through a resampling-based method, that evaluates the stability of clustering. Considering the outcomes of the uniform clustering cumulative distribution function (CDF), delta area plot, and comprehensively, in this consensus cluster analysis on the expression profile of 18 CRGs, three clusters were classified, and all the NSCLC samples can be classified into three clusters according to the outcome of CRGs clusters. Then, differential expression analysis was performed between any two samples on the level of full genomics among the three samples to obtain significant differentially expressed genes. Afterwards, we got three outcomes of differentially expressed genes through sample-to-sample comparison and we intersected the outcomes of differentially expressed genes through Venn diagram, what we obtained is the CRDEGs. During the process, the ‘limma’ package in R software was used to screen the CRDEGs from each subtype for *P*-value = 0.001 and |log2FC| > 2. Then, performing another consensus-cluster analysis on CRDEGs, we got the consensus CRDEGs subtypes.

### Construction of a prognostic score model of CRDEGs and a cuproptosis score model

Using the three CRG subtype clusters, differentially expressed genes of each cluster were taken and intersected using a Wayne diagram. Then, an inter cluster overlap was performed to obtain the expression level of CRDEGs.

The prognostic score model was established on the basis of CRDEGs and filtered by univariate cox analysis through R package. CRDEGs of 1282 samples were treated with multivariate (LASSO)–Cox regression analysis to filter the optimal prognosis-related CRDEGs and acquire the correlation coefficient, HR value, fluctuation range of the HR value of each prognosis-related CRDEGs, on the premise of which, risk score of each patient can be acquired through formula Risk score =$\sum _{1}^{n}coe{f}_{i}\ast {x}_{i}$, where ${x}_{i}$ and $coe{f}_{i}$ represent the expression of each prognosis-related CRDEGs and its corresponding correlation coefficient, respectively. According to the median value of the risk score, the patients were divided into a low-risk and a high-risk group, 53 prognosis-related CRDEGs were filtered to construct the model.

Further, to quantify the cuproptosis patterns of each NSCLC sample, we developed a scoring system to evaluate the cuproptosis pattern of every NSCLC patient: the cuproptosis signature, and we termed this model the cuproptosis score model. The CRDEGs were subjected to univariate Cox regression analysis, and the genes that were statistically significant (*P*-value < 0.05) in terms of prognosis were extracted for further analysis. Principal component analysis (PCA) was utilized to evaluate the cuproptosis score. The score of each sample was calculated through the formula cuproptosis score =$\sum (PC{1}_{i}+PC{2}_{i})$, where i represents the expression of cuproptosis-related genes. The advantage of this method lies in concentrating the score in an integrated series of well correlated (positively or negatively) genes in the set while downregulating the weight contributions from genes which do not comply with another gene set.

### CRG- and CRDEG-related enrichment analysis

Functional enrichment analysis represents a bioinformatic approach applied for the exploration of the distribution of genes in specific pathways. This analysis was performed using the BiocManager packages ‘org.Hs.eg.db’, ‘DOSE’, ‘clusterProfiler’ and ‘enrichplot’, gene ontology (GO), and Kyoto Encyclopedia of Genes and Genomes (KEGG), followed by visualization though the ‘colorspace’, ‘stringi’, and ‘ggplot2’ packages in R software. Immune-related single sample gene set enrichment analysis (ssGSEA) and gene set variation analysis (GSVA) were carried out with BiocManager packages ‘limma’, ‘GSEABase’, and ‘GSVA’. The data were converted into images with the ‘ggpubr’ and ‘pheatmap’ packages in R software.

### Survival analysis in CRGs, CRDEGs subpatterns, cuproptosis prognostic model and cuproptosis score model

To assess the clinical significance of CRGs, CRDEGs subpatterns, cuproptosis prognostic model and cuproptosis score model, coupled with the correlation between cuproptosis based patterns and clinical-pathology characteristics and survival outcomes, we collected the NSCLC patients’ clinical information like gender, age, T-stage, grade, and survival time from the online database. Additionally, the differences in the overall survival (OS) among cluster patterns, cuproptosis score high/low groups, and CRDEGs prognostic score high/low risk patterns were evaluated using Kaplan-Meier analysis with the “survival” and “survminer” packages in R software.

### Construction of a nomograph system

Individual patient’s risk assessment of NSCLC was performed with the ‘rms’ and ‘survival’ packages. The personalized scoring was used to generate a nomogram that predicted the 1-, 3-, and 5-years probabilities of progression-free survival in patients using four parameters. A calibration graph was developed that showed the degree of consistency of our model with an ideal model.

### Receiver operating characteristic (ROC) curve, concordance index (C-index) curve, and decision curve analysis (DCA)

The ROC curve is a comprehensive index reflecting the continuous variables of sensitivity and specificity, a larger area under the curve indicates better diagnostic accuracy. The R software packages ‘survival’, ‘survminer’ and ‘timeROC’ were utilized to develop the ROC image. The C-index curve and DCA were also used to assess the accuracy of the prognostic score model of CRDEGs. The packages ‘ggDCA’, ‘rms’, ‘pec’, and ‘survival’ were used for the analysis.

### Infiltrating immune cells and immune microenvironment features analysis

Immune function and immune infiltration files were downloaded from MSigdb database and Tumor Immune Estimation Resource database (TIMER), BiocManager packages ‘limma’, ‘GSEABase’, ‘GSVA’, and the R packages ‘reshape2’, ‘ggplot2’, ‘ggpubr’, and ‘pheatmap’ were adopted to draft immune-related images. The ESTIMATE (Estimation of Stromal and Immune cells in Malignant Tumor tissues using Expression data) algorithm score was used for calculating the properties of the tumor microenvironment (TME) of the high/low groups of the prognostic score model employed to assess the distinction between these two groups.

### Assessment of immunotherapy sensitivity in prognostic model and recommended therapy in cuproptosis score model

Packages ‘limma’, ‘ggpubr’, ‘pRRophetic’, and ‘ggplot2’ were utilized to carry out drug sensitivity analysis to estimate the level of the half-maximal inhibitory concentrations (IC50) of the low/high risk groups of the prognostic score model. As for the cuproptosis scorer model, immune therapy analysis was implemented on the basis of data acquired from The Cancer Immunity Database (TCIA). The correlation of immune checkpoints CTLA4 and PD-1 in the cuproptosis score high/low groups with immunotherapy sensitivity was exhibited through violin chart drafted by ‘ggpubr’ package in R software.

### Statistical analysis

All statistical analyses were performed with R software, version 4.2.1, and software perl. The Wilcoxon signed-rank test was conducted to determine the expression distinction of CRGs among normal and tumor tissues. The Kaplan-Meier method was utilized to perform survival analysis. The correlation of expression of CRGs with immune cells and immune checkpoints were calculated by Spearman analysis. The PCA method and lasso cox regression analysis were used to build the two models.

## Results

### Clinical features and mutations at the genetic transcription level of CRGs

**Fig. 1:**
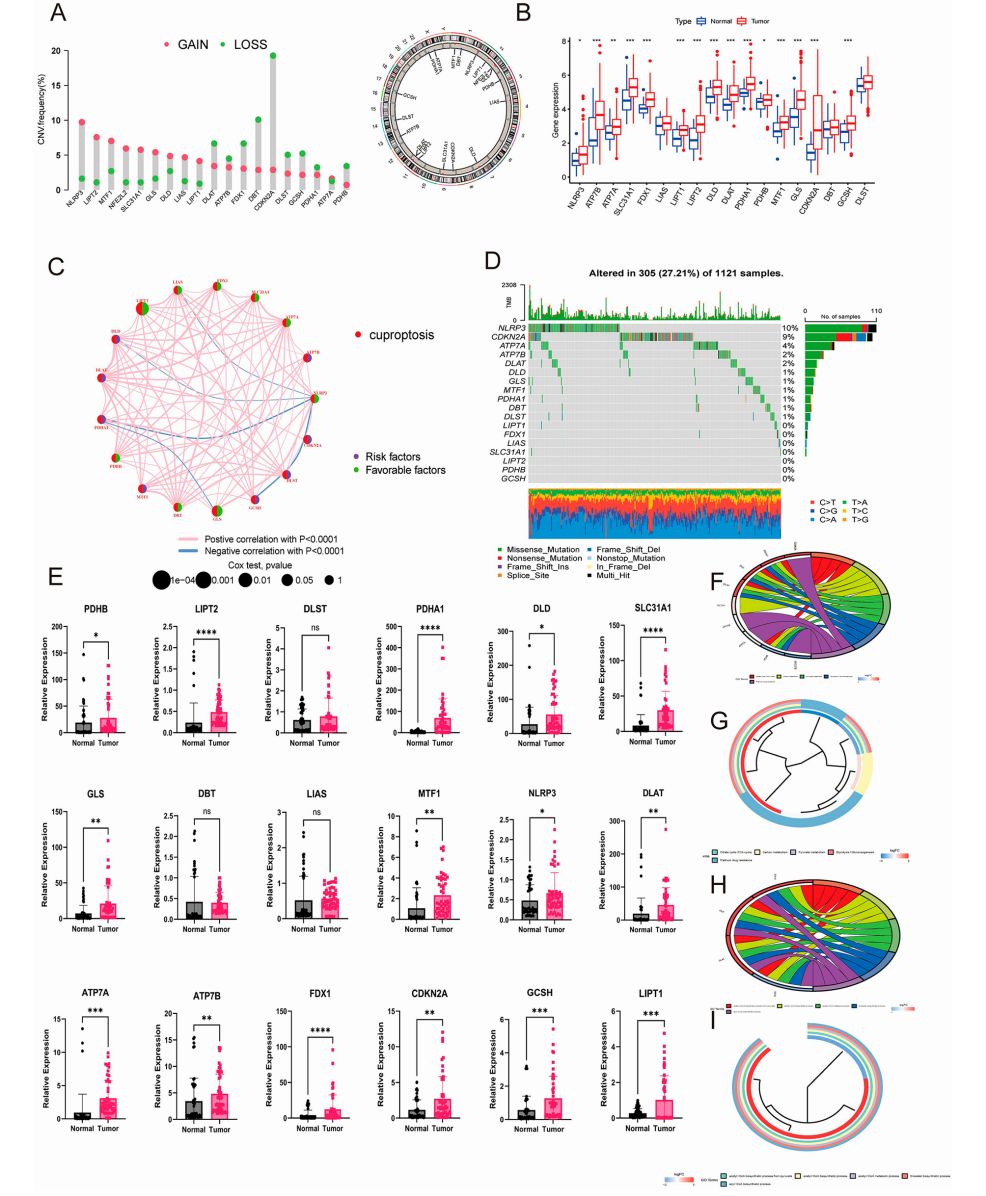
Transcriptional variance and expression of CRGs, and experimental validation and functional enrichment of CRGs in NSCLC. (**A**) Frequency and location of the copy number variation (CNV); (**B**) Variance of the expression of 18 CRGs from TCGA database in the normal and tumor tissues; (**C**) Prognosis-related network of expression of18 CRGs from TCGA database; (**D**) Tumor mutation burden (TMB) of 18 CRGs from TCGA database; (**E**) PCR validation of the CRGs expression level of the tumor and normal tissues; (**F**, **G**) KEGG functional enrichment of CRGs; (**H**, **I**) GO functional enrichment of CRGs (*P < 0.05; ** *P* < 0.01; and *** *P* < 0.001; Ns, not significant). CRGs, cuproptosis-related genes; NSCLC, non-small-cell lung cancer; CNV, copy number variation; TMB, tumor mutation burden; KEGG, Kyoto Encyclopedia of Genes and Genomes; GO, gene ontology

The expression levels of 18 CRGs were acquired from the TCGA dataset and the GEO (GSE42127) dataset, which was used to identify the differential expression of the CRGs between normal and tumor tissues (Fig. 1). All the CRGs were more highly expressed in tumor tissues than in normal tissues; the differences in 15 of these CRGs were statistically significant (*P* < 0.05, Fig. 1B). Experimental validation of the expression in tumor and normal tissues and cell lines was carried out by qRT-PCR (Fig. 1E and Figure S1A). A prognostic correlation network was developed through correlation analysis, which suggested that LIPT1 (*P* < 0.01) and GLS (*P* < 0.05) were probably prognosis-related CRGs (Fig. 1C). Additionally, the CRGs’ CNV and TMB were also estimated (Fig. 1A and D). CDKN2A had the highest CNV frequency and the second highest mutation burden; NLRP3 was associated with the highest TMB.

The clinical survival data and CRG expression from the TCGA and GEO datasets were analyzed to evaluate the correlation of the overall survival rate with the CRGs expression level. The differences in ATP7A, CDKN2A, DLAT, DLST, GLS, LIPT1, and SLC31A CRGs were statistically significant (Figure S2B). Meanwhile, high expression levels of CDKN2A, DLAT, DLST, and SCL31A1 were linked with worse overall survival (OS) rates, whereas the high expression levels of LIPT1, ATP7A, and GLS showed the opposite trend. Functional enrichment revealed that CRGs were primarily enriched in the tricarboxylic acid cycle (TCA) pathway, carbon metabolism (Fig. 1F, G), and the acetyl-CoA biosynthetic process of the pyruvate pathway (Fig. 1H, I), which corresponds to the findings of previous research [29].

### Identification of CRGs and CRDEGs subtypes

**Fig. 2:**
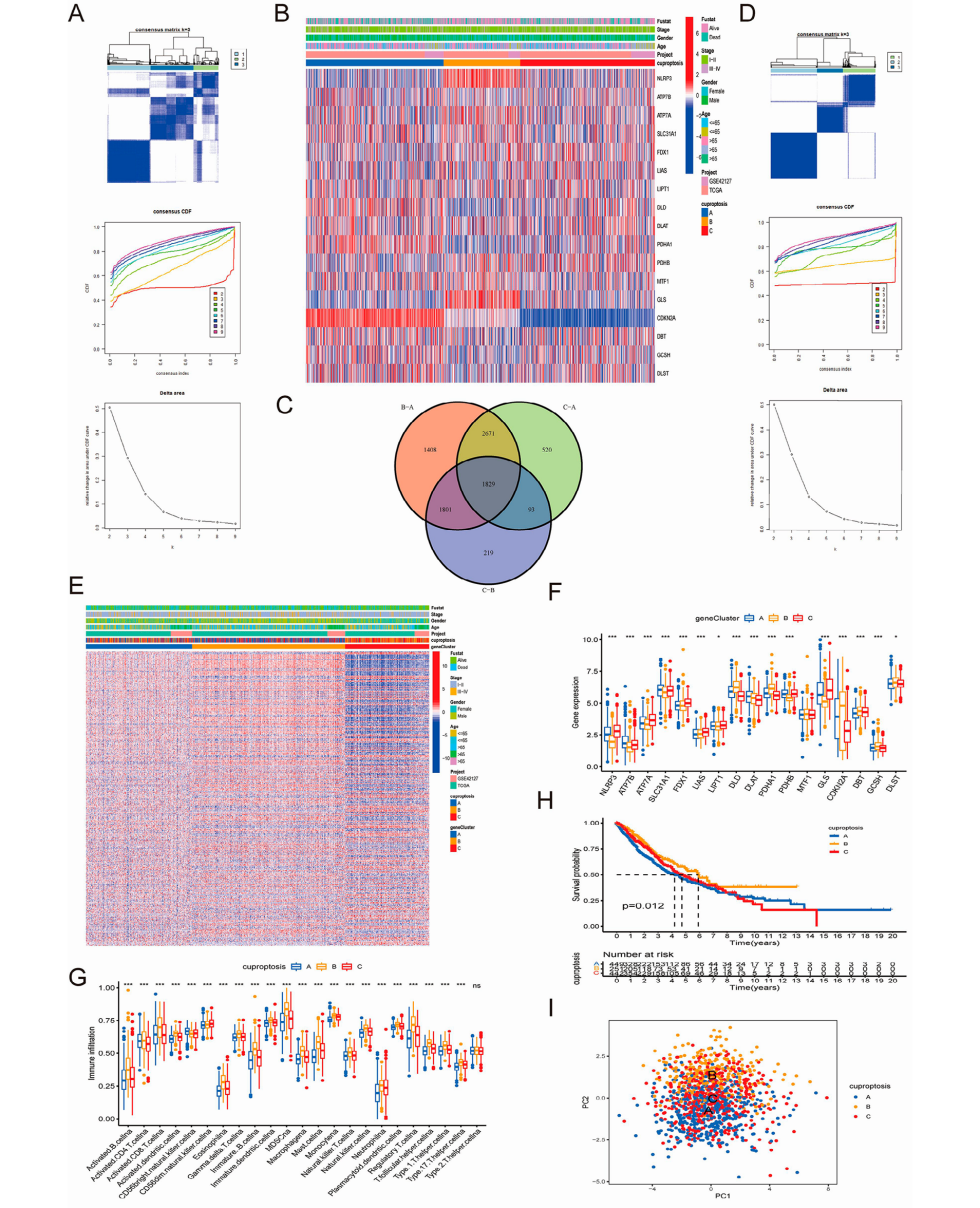
Consensus cluster analyses based on CRGs and CRDEGs. (**A**) Three clusters were divided by consensus cluster analysis coupled with cumulative distribution function plot and delta area plot; (**B**) Heatmap of CRGs expression allied with clinical characteristic of three clusters; (**C**) Venn diagram of CRDEGs; (**D**) Based on CRDEGs, three clusters were divided by consensus cluster analysis coupled with cumulative distribution function and delta area plots; (**E**) Heatmap of CRDEGs expression combined with other clinical features in three subtype clusters; (**F**) Variances in CRGs expression level among three gene clusters; (**G**) Immune infiltration through ssGSEA among the three clusters; (**H**) OS among three clusters. (**I**) Principal component analysis (PCA) of the three clusters. (*P < 0.05; ** *P* < 0.01; and *** *P* < 0.001; Ns, not significant) OS, overall survival; PCA, principal component analysis; ssGSEA, single sample gene set enrichment analysis. CRGs, cuproptosis-related genes. CRDEGs, cuproptosis-related differentially expressed genes; OS, overall survival

To further explore the relationship of CRGs and CRDEGs subtype patterns, the expression level data of the 18 CRGs in 1282 samples were collected from the TCGA and GEO databases. Consensus clustering analysis was then performed, and the optimal k-value was obtained based on the consistent cumulative distribution function and the delta area plots. The k-value was set to range from 1 to 9; and k = 3 generated the best subtype patterns for clustering (Fig. 2A). CRGs expression and other clinical features like stage or survival status are displayed in the heatmap (Fig. 2B). Samples of each cluster that fulfilled the criteria of the adjusted *P*-value = 0.001 and |log2FC| > 2 genes were found to intersect the three subtypes, which were used to determine the CRDEGs, visualized by the Venn diagram displayed in Fig. 2C. Based on the result of these CRDEGs, consensus cluster analysis was again implemented and the an optimal k-value = 3 was also obtained (Fig. 2D). The CRDEG expression and other clinical features such as the stage or survival status are displayed in the heatmap (Fig. 2E), in which suggested that cluster C had lower expression in stage II-IV NSCLC than clusters A and B, which was also lower in all NSCLC samples regardless of the gender. Moreover, the differences in the expression of CRGs among the three clusters were statistically significant (*P* < 0.05) except for that of the MTF1 gene (Fig. 2F).

### Prognostic value, functional enrichment, and immune infiltration analysis of CRGs and CRDEGs subtypes

In the first consensus cluster pattern based on CRGs, the prognosis analysis showed that the OS of cluster B was apparently higher than those of clusters A and C (Fig. 2H). However, the distinction between cluster A and cluster C was not significant, which was reflected in the PCA. The boundary between cluster B and cluster A was clear, whereas those between cluster C and both clusters A and B were not that clear (Fig. 2I). Immune infiltration was performed through ssGSEA (Fig. 2G), in which cluster B had the highest infiltration level among all the clusters, primarily of CD4 + and CD8 + T cells. The representative clinicopathology images, obtained from the TCGA data, shown for each of the three cluster subtypes in Figure S3B, revealed that immune cell infiltration was higher in the tumor nests of cluster B patients than in the other two clusters. Increased CD4 + and CD8 + T cell infiltration has previously been reported to be correlated with better prognosis [30, 31], which may explain why cluster B had the longest OS among the three clusters. GSVA exhibited inter-cluster distinction in the functional enrichment pathways (Figure S3A).

For the second cluster pattern involving genetic modelling, 1829 CRDEGs were selected, which were predominantly correlated with Parkinson’s, Huntington’s, and Alzheimer’s diseases, as well as with a number of other neural degeneration diseases (Figure S4B). These CRDEGs were enriched in the ribonucleoprotein complex biogenesis and mRNA processing pathways (Figure S4A). For this model, the survival analysis indicated that cluster C had higher survival probability than clusters A and B, whereas the difference between cluster A and cluster B was not obvious (Figure S4C).

### Clinical characteristic-related analysis combined with assessment of the tumor mutation burden (TMB) of the prognostic score model and cuproptosis score models in NSCLC

**Fig. 3:**
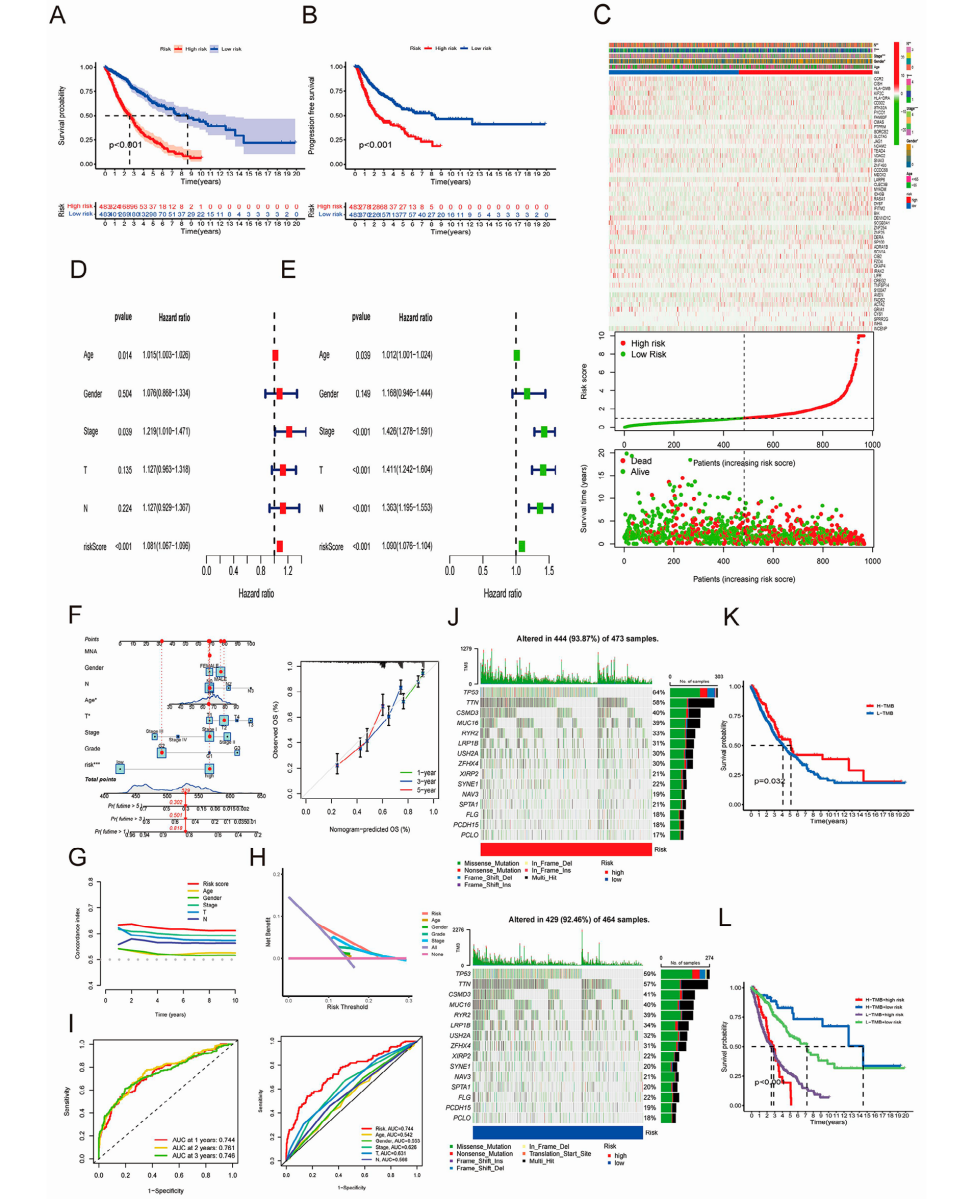
Analysis of a prognostic score model based on CRDEGs. (**A**, **B**) Overall survival (OS) rate and progression free survival (PFS) of the high/low risk groups; (**C**) Heatmap regarding expression of CRDEGs and survival status of the risk high/low groups; (**D**, **E**) Univariate and multivariate cox analysis, green for univariate, and red for multivariate; (**F**) Nomograph and nomograph prediction diagram for the 1-, 3-, and 5-year OS of NSCLC patients; (**G**) C-index curve of risk score and other tumor related clinical characteristics; (**H**) DCA of risk score and other tumor related clinical characteristics; (**I**) ROC and multiROC curve of risk score and other tumor related clinical characteristics; (**J**) TMB situation of the prognostic score model risk high/low group; (**K**, **L**) Survival probability of TMB high/low group and TMB high/low group plus high/low-risk score. (*P < 0.05; ** *P* < 0.01; and *** *P* < 0.001; Ns, not significant). CRDEGs, cuproptosis-related differentially expressed genes; OS, overall survival; PFS, progression free survival; C-index, concordance index; DCA, decision curve analysis; TMB, tumor mutation burden; ROC, receiver operating characteristic curve

A prognostic score model was constructed based on 53 prognosis-related CRDEGs filtered by univariate and multivariate analysis. The prognostic analysis showed that the higher risk score usually indicated a worse OS rate (*P* < 0.001, Fig. 3A) and progression-free survival (PFS) (*P* < 0.001, Fig. 3B). The expression level of the prognosis-related CRDEGs is displayed in the heatmap along with the prognostic high/low-risk score and survival status between the two score groups (Fig. 3C). The status “dead” significantly increased in the high score risk group. Both univariate and multivariate Cox analyses indicated that the prognostic risk score was an independent prognostic factor in NSCLC (Fig. 3D, E). Applying the prognostic model and clinical score parameters, we established a nomogram to estimate the 1-, 3-, and 5-year OSs of NSCLC patients (Fig. 3F). The consistency of the calibration curves of this nomogram determines the accuracy of the predicted values. This nomograph may be advantageous in predicting the clinical prognosis and can be used as a clinical decision-making tool in the future. The C-index curve revealed that our prognostic model had a higher concordance index than the other clinical factors (Fig. 3G). Similarly, the DCA and ROC curve also confirmed this outcome. Therefore, our CRDEGs prognostic model was the most precise method of predicting the NSCLC prognosis (Fig. 3H and I). The higher risk score group had higher mutation frequency than the lower risk group (Fig. 3J), but the difference between the two groups was not statistically significant (*P* = 0.81, Figure S2A). However, the OSs of the TMB groups were significantly different. Surprisingly, better OS was found in the high-TMB group (Fig. 3K). Nevertheless, when the risk score was considered, a higher risk was always associated with worse OS as compared with the low-risk group regardless of whether the TMB was high or low (Fig. 3L).

**Fig. 4:**
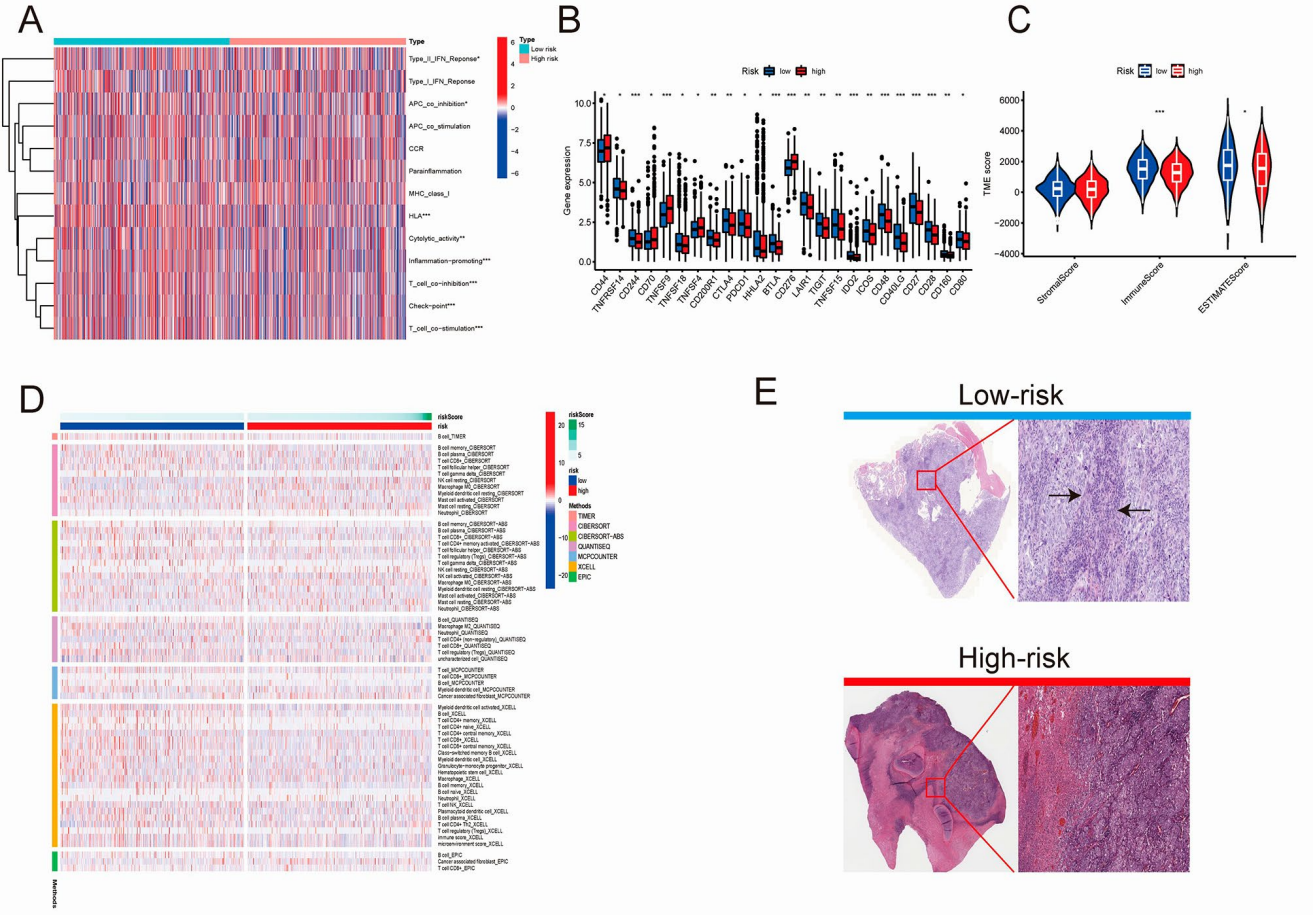
Analysis of the CRG and CRDEG clusters. (**A**) Cuproptosis score among three CRGs clusters and CREDGs clusters; (**B**) Survival probability of high/low group in cuproptosis score model NSCLC patients; (**C**) Correlation of survival period, survival status, and NSCLC major mutant gene EGFR with two groups in cuproptosis score model; (**D**) Survival curve of NSCLC patients from different stages; (**E**) TMB among the two groups of cuproptosis score models and the three gene clusters; (**F**) TMB in the cuproptosis score model high/low groups; (**G**, **H**) Survival probability in the high-/low-TMB group and the high/low-TMB group plus the high/low cuproptosis score; (**I**) Sankey diagram of the CRGs and CRDEGs subtype clusters, the cuproptosis score group, and the survival status. (*P < 0.05; ** *P* < 0.01; and *** *P* < 0.001; Ns, not significant). TMB, tumor mutation burden. EGFR, epidermal growth factor receptor; NSCLC, non-small-cell lung cancer

А cuproptosis score model was established through univariate Cox analysis of CRDEGs processed by PCA. The cuproptosis score was statistically significant among both CRGs subtype clusters and CRDEGs subtype clusters (Fig. 4A). The survival probability of the cuproptosis score model indicated that the high score group correlated with better OS (*P* < 0.001, Fig. 4B and C). The same tendency was found in patients with different cancer stages (Fig. 4D). There was a statistically significant distinction between the high-cuproptosis-score group (87.93%) the low-cuproptosis-score group (98.87%) (Fig. 4F). The low-cuproptosis-score group showed an apparent TMB upregulation. Ranked from high to low, the TMB in the gene clusters was as follows: cluster B, cluster A, and cluster C (Fig. 4E). The OS of the high-TMB group was longer than that of the low-TMB group, when the cuproptosis score was taken into consideration. The high-TMB group plus the high-cuproptosis group was correlated with better OS than the high-TMB group plus low-cuproptosis group (*P* = 0.032, Fig. 4G; *P* = 0.007, Fig. 4H). Therefore, it is reasonable to speculate that a high cuproptosis score is linked with a better probability of survival. The difference in the cuproptosis score is related to different clinical features. The survival probability in the high-score cuproptosis group was higher than that in the low-score cuproptosis group (Fig. 4C). EGFR is one of the most essential receptors in the progress of NSCLC tumorigenesis and tumor development [32]. A low cuproptosis score was related with a higher level of EGFR expression, which may result in worse OS in NSCLC (Fig. 4C). The Sankey diagram of CRGs subtype clusters, CRDEGs subtype clusters, cuproptosis score group, and survival status is presented in Fig. 4I.

### TME and immune treatment sensitivity of the prognostic score model and the cuproptosis score model in NSCLC

**Fig. 5:**
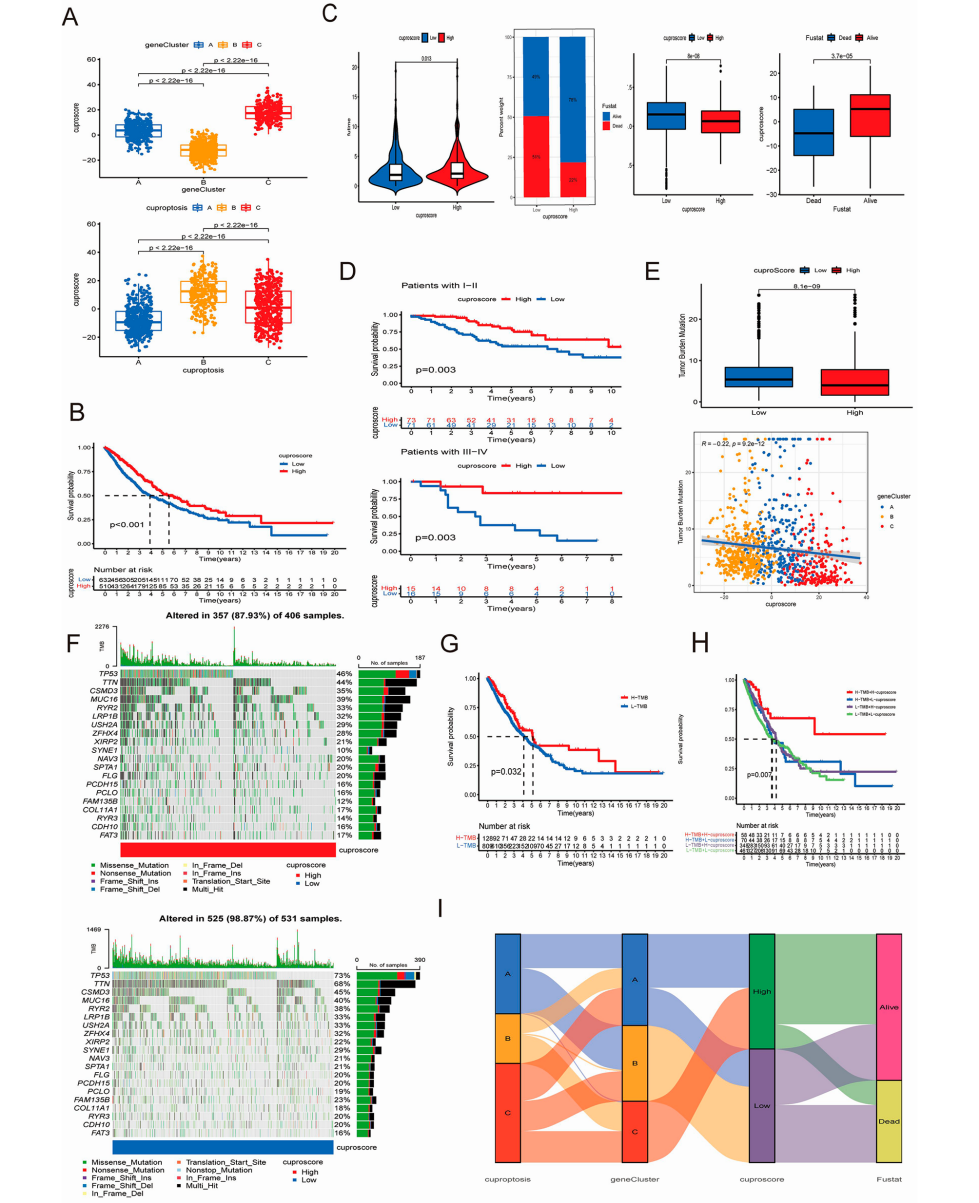
Immune-related analysis of the prognostic score model high/low risk groups. (**A**) Immune function pathway analysis of prognostic score model; (**B**) Immune checkpoint analysis of the prognostic score model; (**C**) TME score of high/low risk group; (**D**) Heatmap of immune cell infiltration of the two groups; (**E**) Representative image of HE staining from the TCGA database showing the degree of immune cell infiltration of the two groups. (*P < 0.05; ** *P* < 0.01; and *** *P* < 0.001; Ns, not significant). TME, tumor microenvironment. TCGA, The Cancer Genome Atlas; HE, hematoxylin and eosin staining

Based on the prognostic model, the immune function analysis (Fig. 5A) showed that compared with the high-risk score group, the low-risk group is enriched mainly in the cytolytic, T-cell stimulation, and inflammation-promoting pathways. A high level of T-cell immune infiltration may be correlated with the better prognosis in the low-risk NSCLC group. Immune checkpoint analysis (Fig. 5B) revealed that basically all the checkpoints were upregulated in the low-risk group except for CD44, TNFSF4/9, and CD276 (marker of cancer stem cell) [33]. Based on the TME score, we conclude that a distinction existed between the high/low risk groups in terms of immune and estimate scores (Fig. 5C). Immune cell infiltration analysis showed that CD4^+^T and CD8^+^T cells were enriched mainly in the low-risk score group, whereas macrophage M0- and cancer-associated fibroblast cells were highly infiltrated in the high-risk model group (Fig. 5D), which corresponds to the outcomes of the survival and PFS analysis. Representative clinicopathology images from the TCGA data showed that the low-risk score group had higher immune cell infiltration (Fig. 5E). Through drug sensitivity tests we selected four potential reagents which might be correlated with the better efficacy in the high-risk group of prognostic score model while another four reagents may be correlated with the same effect in the low-risk group of prognostic score model. (Figure S5A).

**Fig. 6:**
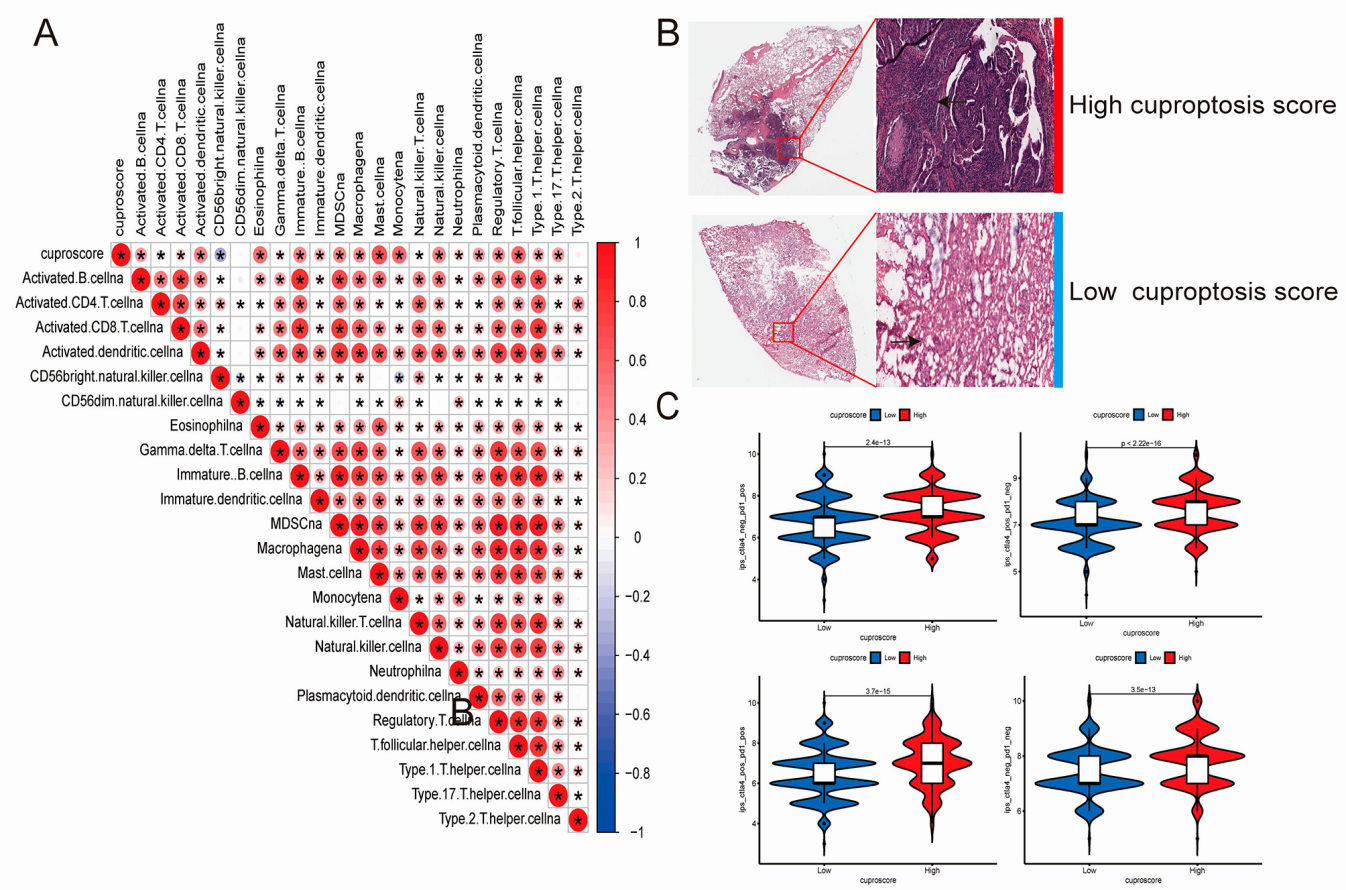
Clinical characteristics and immune correlation analysis of the cuproptosis score model. (**A**) Immune infiltration correlation analysis network; (**B**) Representative image of HE staining from the TCGA database showing the immune cell infiltration in the two high-score cuproptosis groups from TCGA; (**C**) Immunotherapy efficacy score in the two groups. (*P < 0.05; ** *P* < 0.01; and *** *P* < 0.001; Ns, not significant). TCGA, The Cancer Genome Atlas; HE, hematoxylin and eosin

**Fig. 7:**
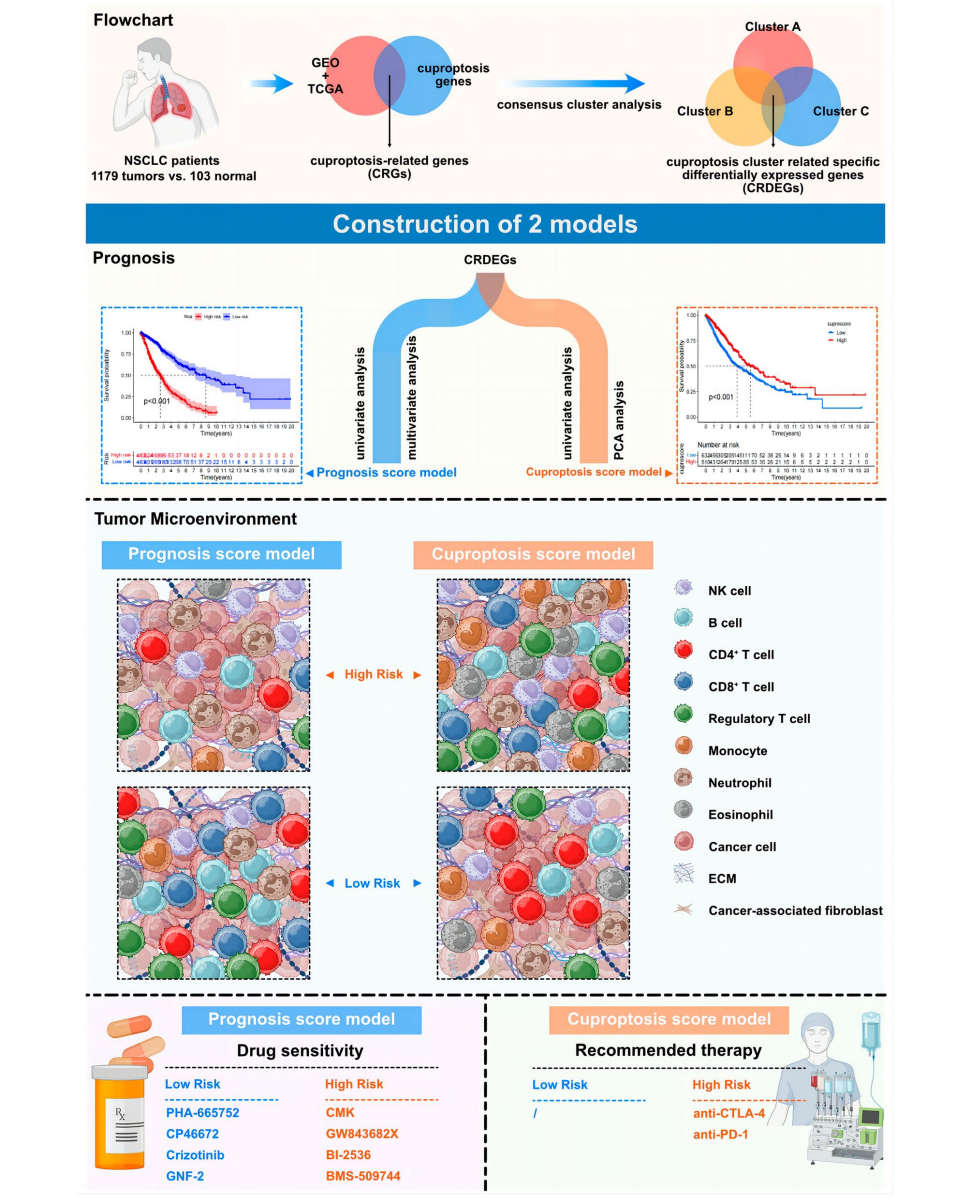
Overall graphical summary and brief flow chart of the study

The correlation diagram (Fig. 6A) of the cuproptosis group revealed that a higher cuproptosis score was positively associated with the activation of B cells, dendritic cells, eosinophilia, immature B cells, MDSC, macrophages, mast cells, monocytes, natural killer cells, plasmacytoid dendritic cells, regulatory T cells, T follicular helper cells, type 1 T-helper cells, and type 17 T-helper cells. Representative clinicopathology images were acquired from the TCGA data which showed that the high-cuproptosis-score group had greater immune cell infiltration (Fig. 6B). Regardless of whether the immune target was CTLA4- and PD1-positive or -negative, better immune effect was observed in the high-cuproptosis group than in the low-cuproptosis group (Fig. 6C). These results supported our hypothesis and provide solid evidence for the rationality of constructing prognostic and cuproptosis score models (Fig. 7).

## Discussion

Copper ions have recently been shown to be involved in a cell death pathway [34, 35], a process that is linked with tumorigenesis, metastasis, and immune function in cancer [17, 19]. It is possible that the knockdown of copper chaperone antioxidant-1 inhibits copper-stimulated proliferation of NSCLC [36] and that the copper transporter ATP7A is correlated with platinum chemotherapy resistance in NSCLC [37]. Moreover, autophagy suppression contributes to disulfiram/copper-induced apoptosis in NSCLC cells [38]. Key targets of copper homeostasis in humans and mammals include ceruloplasmin, CTR1 (known as SLC31A1) as the major protein carrier of exchangeable copper in plasma, and ion transporters associated with cellular copper uptake, cytosolic metal chaperones and cytosolic mitochondrial metal chaperones target copper insertion into metalloenzymes and copper-dependent ATP enzymes like ATP7A and ATP7B [16], which have copper export and metal chaperone functions. Metallothionein 1 (MT1) and MT2 are two of three thiol-rich proteins that bind with high affinity to a variety of copper ions. At the same time, copper is also a dynamic signaling metal and metal allosteric regulator, such as copper-dependent phosphodiesterase 3B (PDE3B) in lipolysis, mitogen-activated protein kinase kinase 1 (MEK1) in cell growth and proliferation and MEK2, the kinases ULK1 and ULK2 in autophagy [39, 40]. Cell proliferation that relies on copper is called copper hyperplasia, which corresponds to this article about copper death. These enzymes may have some interactions with FDX1 signaling, which may be a focus of future research. Therefore, it is reasonable to speculate that there is a link between cuproptosis and NSCLC. We undertook an analysis of a cuproptosis scoring model based on different cuproptosis subtypes and a prognostic score model of CRDEGs to further explore the clinical significance of cuproptosis and the mechanisms of its interactions with the immune microenvironment. These two models improve the existing understanding of cuproptosis in NSCLC and may provide an innovative guidance for clinical practice.

Eighteen CRGs were classified into three subtypes by consensus clustering. Survival analysis results suggested that subtype B had a better prognosis than subtypes A and C, and that the different subtypes had obvious enrichment differences in certain pathways. Immune infiltration pathways with distinct enrichment discrepancy were also evident among the three subtypes. The greatest differences among the three subtype clusters were observed in the T cell-related pathways. Enrichment analysis of the CRGs showed that they were enriched mainly in the TCA pathway, which is in agreement with the latest research evidence [41, 42]. Three gene clusters were developed after consensus clustering was performed on CRDEGs. Cluster C had a better prognosis than clusters A and B, implying that the subtypes of CRGs and CRDEGs may be associated with the clinical characteristics of NSCLC patients.

The cuproptosis score model was established based on the prognosis-related genes acquired by univariate Cox analysis of CRDEGs to determine the cuproptosis value of each sample. Higher cuproptosis score indicated a better OS rate, even within patients at different cancer stages. The low-cuproptosis-score group had a higher TMB than the high-cuproptosis group. This is an interesting point because in our earlier analysis, the OSs of different TMB groups were significantly different. Surprisingly, a better OS was found in the high-TMB group. It is possible that high TMB may improve the recognition of tumor cells by the immune system, facilitating the attack and elimination of tumor cells by immune cells. High mutational burden may also lead to more adaptive variants in tumor cells and the production of more aggressive, drug-resistant subclones. The intersection of high-/low-cuproptosis-score and mutation samples was obtained in this cuproptosis score model the mutation burden survival curve analysis, requires the intersection of high/low cuproptosis score samples and mutation samples,. The number of intersection samples in the high-cuproptosis score group was significantly lower than that in the low-cuproptosis-score group, which might have caused bias in analysis. However, when survival was analyzed with the cuproptosis score combined with the mutation rate, the prognosis of the high-cuproptosis-score group was better than that of the low-cuproptosis-score group, regardless of the combination of high/low TMB. The results of the ssGSEA and immune correlation analyses indicated that the cuproptosis score was highly positively correlated with the levels of mast cells, monocytes, and follicular T-helper cells, a specialized CD4^+^ T cell type primarily found in the tonsils [43]. Meanwhile, the NSCLC-related molecule EGFR [44] was upregulated in the low-cuproptosis group.

Immunotherapy is essential for limiting tumorigenesis and progression [45], and thus, regardless of the immune molecules status, the high-cuproptosis-score group showed better efficacy than the lower group. This result indicates that cuproptosis is likely to be involved in the changes of tumor development progress and TME.

Previous research on CRDEGs enrichment analysis has been concentrated on neurodegeneration diseases such as Alzheimer’s disease [46]. The filtering of CRDEGS in our univariate and multivariate Cox analyses yielded 53 prognosis-related CRDEGs, which were then screened for the development of this novel model. The low-risk group had better prognosis regardless of OS or PFS, which is directly opposite to the results obtained in the cuproptosis score model. Both univariate and multivariate Cox analyses showed that the risk score of the prognostic model was an independent prognostic factor. Contrary to the cuproptosis score system, in the prognostic score model, the high-risk score group had higher TMB than the low-risk group. Immune cell infiltration analysis revealed that CD4^+^T and CD8^+^T cells were enriched mainly in the low-risk score group, whereas macrophage M0 and cancer-associated fibroblast cells were highly infiltrated the high-risk model group. The distinction in TME may lead to the difference in the prognosis between the two groups. This provides evidence that CD4^+^T cells and CD8^+^T cells are correlated with better prognosis in NSCLC [47, 48]. Drug sensitivity testing results has demonstrated merely correlation relationships between eight agents and high-/low-risk groups, four of which might be related with higher sensitivities in the high-risk group and the other four agents might be related with better efficacy in low-risk group.

Considering that the two models were established from different aspects and through different methods, differences in the results were expected. However, the subsequent clinicopathological analysis, prognostic analysis, and immune microenvironment analysis of NSCLC through both models supports diverse ideas and approaches to guide the clinical screening, diagnosis and treatment of NSCLC. There are limitations in this research that have to be considered. First, all the data were obtained from public data sources, and such retrospective data are vulnerable to selection bias, which might have interfered with the accuracy of our analysis. Additionally, for further explorations of cuproptosis in NSCLC, more clinical features and data should be taken into consideration, which were lacking in the publicly accessible databases. Finally, more experimental assays are required to validate more comprehensively the bioinformatics analysis results.

## Conclusions

In this study, we systematically constructed two models and performed clinicopathological, prognostic, and immunological analyses to explore the involvement of cuproptosis in tumorigenesis and tumor development in NSCLC. These CRGs and CRDEGs may have the potential to serve as effective immunotherapy and chemotherapy sensitivity biomarkers. Therefore, both the cuproptosis score model and the prognostic model can be clinically significant and may provide novel targets for individualized immunotherapy in the future.

### Electronic supplementary material

Below is the link to the electronic supplementary material.

**Supplementary Material 1: Figure S1.** (A) PCR validation of CRGs expression level among normal cell lines and NSCLC cell lines.

**Supplementary Material 2: Figure S2.** (A) No statistically significant was found high/low groups of prognostic score model in terms of TMB. TMB, tumor mutation burden. (B) Survival probability of seven statistically significant CRGs. TMB, tumor mutation load.

**Supplementary Material 3: Figure S3.** GSVA analysis performed on CRGs. (A) Inter cluster comparison in terms of distinction in function enrichment pathway. (B) Slide image of lymphocyte infiltration of three clusters. CRGs, cuproptosis related genes; GSVA, Gene set variation analysis

**Supplementary Material 4: Figure S4.** Acquisition and functional enrichment of CRDEGs. (A) GO functional enrichment of CRDEGs. (B) KEGG functional enrichment of CRDEGs. (C) Survival curve of three gene clusters. (*p < 0.05; **p < 0.01; ***p < 0.001; Ns, not significant). CRDEGs, cuproptosis related differentially expressed genes; GO, gene ontology; KEGG, Kyoto Encyclopedia of Genes and Genomes

**Supplementary Material 5: Figure S5.** (A) Drug sensitivity of the high/low-groups of the prognostic score model.

**Supplementary Material 6:** Primers

## Data Availability

The datasets generated and analyzed during the present study are available in the TCGA repository (https://portal.gdc.cancer.gov/) and GEO repository (GSE42127) (https://www.ncbi.nlm.nih.gov/).

## Abbreviations

CDF: Cumulative distribution function
CNV: Copy number variation
CRDEGs: Cluster-related specific differentially expressed genes
CRGs: Cuproptosis-related genes
CT: Computed tomography
DLAT: Dihydrolipoamide acetyltransferase
EBUS-TBNA: Endobronchial ultrasound-guided needle aspiration biopsy
EGFR: Epidermal growth factor receptor
ESTIMATE: Estimated Stromal and Immune cells in Malignant Tumor tissues using Expression data
GADPH: Glyceraldehyde-3-phosphate dehydrogenase
GEO: Gene Expression Omnibus
GPX4: Glutathione peroxidase
GSVA: Gene set variation analysis
KEGG: Kyoto Encyclopedia of Genes and Genomes
lncRNAs: Long non-coding RNAs
LUAD: Lung adenocarcinoma
NSCLC: Non-small-cell lung cancer
PCA: Principal component analysis
PD-1: Programmed cell death-1
SCLC: Small-cell lung cancer
TCGA: The Cancer Genome Atlas
TCIA: The Cancer Immunity Database
TIMER: Tumor Immune Estimation Resource database
TMB: Tumor mutation load
TME: Tumor microenvironment

## References

[1] Nasim F, Sabath B, Eapen G (2019). Lung Cancer. Med Clin N Am.

[2] Park K, Vansteenkiste J, Lee K, Pentheroudakis G, Zhou C, Prabhash K, Seto T, Voon P, Tan D, Yang J (2020). Pan-asian adapted ESMO Clinical Practice guidelines for the management of patients with locally-advanced unresectable non-small-cell lung cancer: a KSMO-ESMO initiative endorsed by CSCO, ISMPO, JSMO, MOS, SSO and TOS. Annals Oncology: Official J Eur Soc Med Oncol.

[3] Cronin K, Lake A, Scott S, Sherman R, Noone A, Howlader N, Henley S, Anderson R, Firth A, Ma J (2018). Annual Report to the Nation on the Status of Cancer, part I: national cancer statistics. Cancer.

[4] Lahiri A, Maji A, Potdar PD, Singh N, Parikh P, Bisht B, Mukherjee A, Paul MK (2023). Lung cancer immunotherapy: progress, pitfalls, and promises. Mol Cancer.

[5] Borghaei H, Paz-Ares L, Horn L, Spigel D, Steins M, Ready N, Chow L, Vokes E, Felip E, Holgado E (2015). Nivolumab versus Docetaxel in Advanced Nonsquamous Non-small-cell Lung Cancer. N Engl J Med.

[6] Johnson M, Garassino MC, Mok T, Mitsudomi T (2022). Treatment strategies and outcomes for patients with EGFR-mutant non-small cell lung cancer resistant to EGFR tyrosine kinase inhibitors: focus on novel therapies. Lung Cancer.

[7] Siegel R, Miller K, Fuchs H, Jemal A (2021). Cancer statistics, 2021. Cancer J Clin.

[8] Leiter A, Veluswamy R, Wisnivesky J (2023). The global burden of lung cancer: current status and future trends. Nat Reviews Clin Oncol.

[9] Keek S, Kayan E, Chatterjee A, Belderbos J, Bootsma G, van den Borne B, Dingemans A, Gietema H, Groen H, Herder J (2022). Investigation of the added value of CT-based radiomics in predicting the development of brain metastases in patients with radically treated stage III NSCLC. Therapeutic Adv Med Oncol.

[10] Kandathil A, Subramaniam R (2022). FDG PET/CT for primary staging of Lung Cancer and Mesothelioma. Semin Nucl Med.

[11] Fulda S (2013). The mechanism of necroptosis in normal and cancer cells. Cancer Biol Ther.

[12] Ashkenazi A, Salvesen G (2014). Regulated cell death: signaling and mechanisms. Annu Rev Cell Dev Biol.

[13] Strasser A, Vaux DL (2020). Cell death in the origin and treatment of Cancer. Mol Cell.

[14] Zhang C, Liu X, Jin S, Chen Y, Guo R (2022). Ferroptosis in cancer therapy: a novel approach to reversing drug resistance. Mol Cancer.

[15] Santagostino S, Assenmacher C, Tarrant J, Adedeji A, Radaelli E (2021). Mechanisms of regulated cell death: current perspectives. Vet Pathol.

[16] Tsvetkov P, Coy S, Petrova B, Dreishpoon M, Verma A, Abdusamad M, Rossen J, Joesch-Cohen L, Humeidi R, Spangler R (2022). Copper induces cell death by targeting lipoylated TCA cycle proteins. Sci (New York NY).

[17] Ge E, Bush A, Casini A, Cobine P, Cross J, DeNicola G, Dou Q, Franz K, Gohil V, Gupta S (2022). Connecting copper and cancer: from transition metal signalling to metalloplasia. Nat Rev Cancer.

[18] Liu Y, Bager C, Willumsen N, Ramchandani D, Kornhauser N, Ling L, Cobham M, Andreopoulou E, Cigler T, Moore A (2021). Tetrathiomolybdate (TM)-associated copper depletion influences collagen remodeling and immune response in the pre-metastatic niche of breast cancer. NPJ Breast cancer.

[19] Voli F, Valli E, Lerra L, Kimpton K, Saletta F, Giorgi F, Mercatelli D, Rouaen J, Shen S, Murray J (2020). Intratumoral Copper modulates PD-L1 expression and influences Tumor Immune Evasion. Cancer Res.

[20] Sha S, Si L, Wu X, Chen Y, Xiong H, Xu Y, Liu W, Mei H, Wang T, Li M (2022). Prognostic analysis of cuproptosis-related gene in triple-negative breast cancer. Front Immunol.

[21] Zhou Y, Shu Q, Fu Z, Wang C, Gu J, Li J, Chen Y, Xie M (2022). A novel risk model based on cuproptosis-related lncRNAs predicted prognosis and indicated immune microenvironment landscape of patients with cutaneous melanoma. Front Genet.

[22] Mo X, Hu D, Yang P, Li Y, Bashir S, Nai A, Ma F, Jia G, Xu M (2022). A novel cuproptosis-related prognostic lncRNA signature and lncRNA MIR31HG/miR-193a-3p/TNFRSF21 regulatory axis in lung adenocarcinoma. Front Oncol.

[23] Song Q, Zhou R, Shu F, Fu W (2022). Cuproptosis scoring system to predict the clinical outcome and immune response in bladder cancer. Front Immunol.

[24] Ma S, Zhu J, Wang M, Zhu J, Wang W, Xiong Y, Jiang R, Seetharamu N, Abrão F, Puthamohan V (2022). A cuproptosis-related long non-coding RNA signature to predict the prognosis and immune microenvironment characterization for lung adenocarcinoma. Translational lung cancer Res.

[25] Li Q, Wang T, Zhou Y, Shi J. Cuproptosis in lung cancer: mechanisms and therapeutic potential. *Molecular and cellular biochemistry* 2023. 10.1007/s11010-023-04815-y 37480450

[26] Ma C, Li F, Gu Z, Yang Y, Qi Y (2023). A novel defined risk signature of cuproptosis-related long non-coding RNA for predicting prognosis, immune infiltration, and immunotherapy response in lung adenocarcinoma. Front Pharmacol.

[27] Xiaona X, Liu Q, Zhou X, Liang R, Yang S, Xu M, Zhao H, Li C, Chen Y, Xueding C (2023). Comprehensive analysis of cuproptosis-related genes in immune infiltration and prognosis in lung adenocarcinoma. Comput Biol Med.

[28] Ling X, Zhang L, Fang C, Liang H, Zhu J, Ma J (2023). Development of a cuproptosis-related signature for prognosis prediction in lung adenocarcinoma based on WGCNA. Translational lung cancer Res.

[29] Duan W, He R (2022). Cuproptosis: copper-induced regulated cell death. Sci China Life Sci.

[30] Eberst G, Vernerey D, Laheurte C, Meurisse A, Kaulek V, Cuche L, Jacoulet P, Almotlak H, Lahourcade J, Gainet-Brun M (2022). Prognostic value of CD4 + T lymphopenia in non-small cell lung Cancer. BMC Cancer.

[31] Zhang G, Liu A, Yang Y, Xia Y, Li W, Liu Y, Zhang J, Cui Q, Wang D, Liu X (2022). Clinical predictive value of naïve and memory T cells in advanced NSCLC. Front Immunol.

[32] Lin A, Wei T, Meng H, Luo P, Zhang J (2019). Role of the dynamic tumor microenvironment in controversies regarding immune checkpoint inhibitors for the treatment of non-small cell lung cancer (NSCLC) with EGFR mutations. Mol Cancer.

[33] Wang C, Li Y, Jia L, Kim J, Li J, Deng P, Zhang W, Krebsbach P, Wang C (2021). CD276 expression enables squamous cell carcinoma stem cells to evade immune surveillance. Cell Stem Cell.

[34] Gao W, Huang Z, Duan J, Nice E, Lin J, Huang C (2021). Elesclomol induces copper-dependent ferroptosis in colorectal cancer cells via degradation of ATP7A. Mol Oncol.

[35] Guo H, Ouyang Y, Yin H, Cui H, Deng H, Liu H, Jian Z, Fang J, Zuo Z, Wang X (2022). Induction of autophagy via the ROS-dependent AMPK-mTOR pathway protects copper-induced spermatogenesis disorder. Redox Biol.

[36] Cai H, Peng F (2013). Knockdown of copper chaperone antioxidant-1 by RNA interference inhibits copper-stimulated proliferation of non-small cell lung carcinoma cells. Oncol Rep.

[37] Li Z, Qiu M, Zeng Z, Luo H, Wu W, Wang F, Wang Z, Zhang D, Li Y, Xu R (2012). Copper-transporting P-type adenosine triphosphatase (ATP7A) is associated with platinum-resistance in non-small cell lung cancer (NSCLC). J Translational Med.

[38] Wu X, Xue X, Wang L, Wang W, Han J, Sun X, Zhang H, Liu Y, Che X, Yang J (2018). Suppressing autophagy enhances disulfiram/copper-induced apoptosis in non-small cell lung cancer. Eur J Pharmacol.

[39] Xie J, Yang Y, Gao Y, He J (2023). Cuproptosis: mechanisms and links with cancers. Mol Cancer.

[40] Chen L, Min J, Wang F (2022). Copper homeostasis and cuproptosis in health and disease. Signal Transduct Target Therapy.

[41] Tang D, Chen X, Kroemer G (2022). Cuproptosis: a copper-triggered modality of mitochondrial cell death. Cell Res.

[42] Cobine P, Brady D (2022). Cuproptosis: Cellular and molecular mechanisms underlying copper-induced cell death. Mol Cell.

[43] Xu F, Zhang H, Chen J, Lin L, Chen Y (2020). Immune signature of T follicular helper cells predicts clinical prognostic and therapeutic impact in lung squamous cell carcinoma. Int Immunopharmacol.

[44] Salama M, Liu M, Clarke C, Espaillat M, Haley J, Jin T, Wang D, Obeid L, Hannun Y (2019). PKCα is required for Akt-mTORC1 activation in non-small cell lung carcinoma (NSCLC) with EGFR mutation. Oncogene.

[45] Osmani L, Askin F, Gabrielson E, Li Q (2018). Current WHO guidelines and the critical role of immunohistochemical markers in the subclassification of non-small cell lung carcinoma (NSCLC): moving from targeted therapy to immunotherapy. Sem Cancer Biol.

[46] Lai Y, Lin C, Lin X, Wu L, Zhao Y, Lin F (2022). Identification and immunological characterization of cuproptosis-related molecular clusters in Alzheimer’s disease. Front Aging Neurosci.

[47] Talebian Yazdi M, van Riet S, van Schadewijk A, Fiocco M, van Hall T, Taube C, Hiemstra P, van der Burg S (2016). The positive prognostic effect of stromal CD8 + tumor-infiltrating T cells is restrained by the expression of HLA-E in non-small cell lung carcinoma. Oncotarget.

[48] Xia L, Wang H, Sun M, Yang Y, Yao C, He S, Duan H, Xia W, Sun R, Yao Y (2021). Peripheral CD4 T cell signatures in predicting the responses to anti-PD-1/PD-L1 monotherapy for Chinese advanced non-small cell lung cancer. Sci China Life Sci.

