# Supplementary figures and images for: A cuproptosis score model and prognostic score model can evaluate clinical characteristics and immune microenvironment in NSCLC
Source: Cancer Cell Int. 2024 Feb 10;24:68. doi: 10.1186/s12935-024-03267-8 (PMC10859031; doi:10.1186/s12935-024-03267-8)

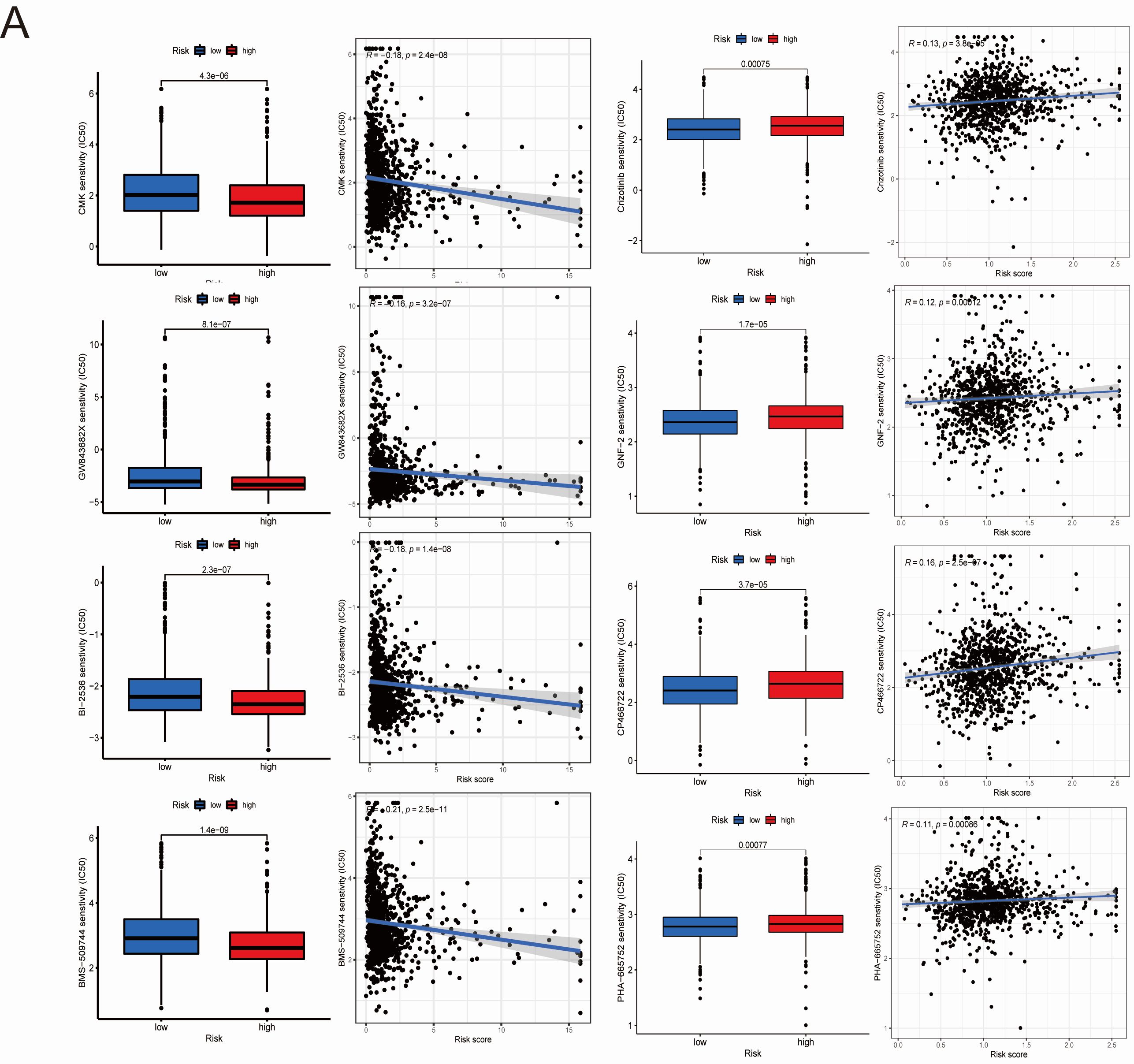

Supplement: Supplementary file 1 — Supplementary Material 1: Figure S1. (A) PCR validation of CRGs expression level among normal cell lines and NSCLC cell lines. [file 12935_2024_3267_MOESM1_ESM.jpg]

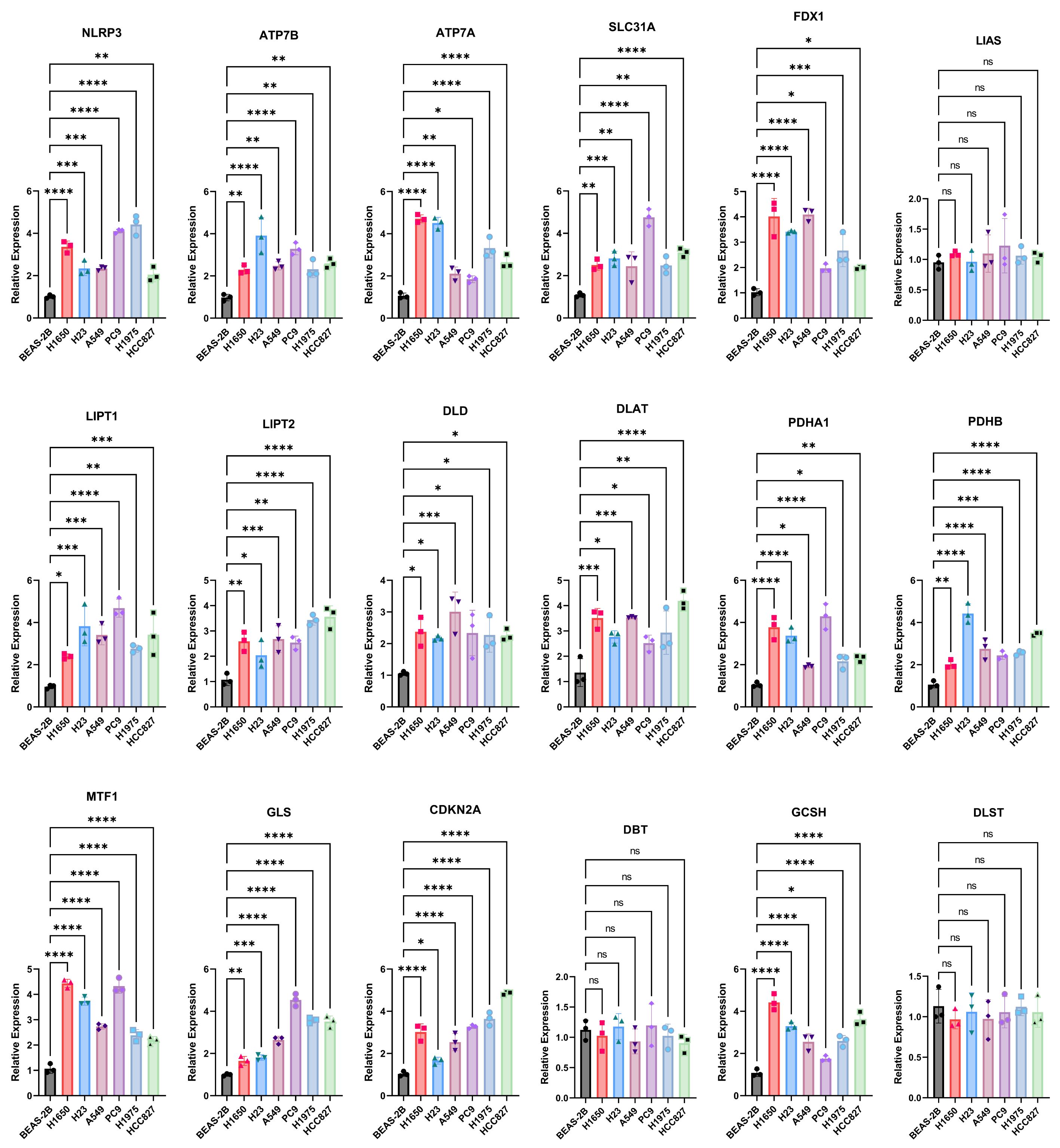

Supplement: Supplementary file 2 — Supplementary Material 2: Figure S2. (A) No statistically significant was found high/low groups of prognostic score model in terms of TMB. TMB, tumor mutation burden. (B) Survival probability of seven statistically significant CRGs. TMB, tumor mutation load. [file 12935_2024_3267_MOESM2_ESM.jpg]

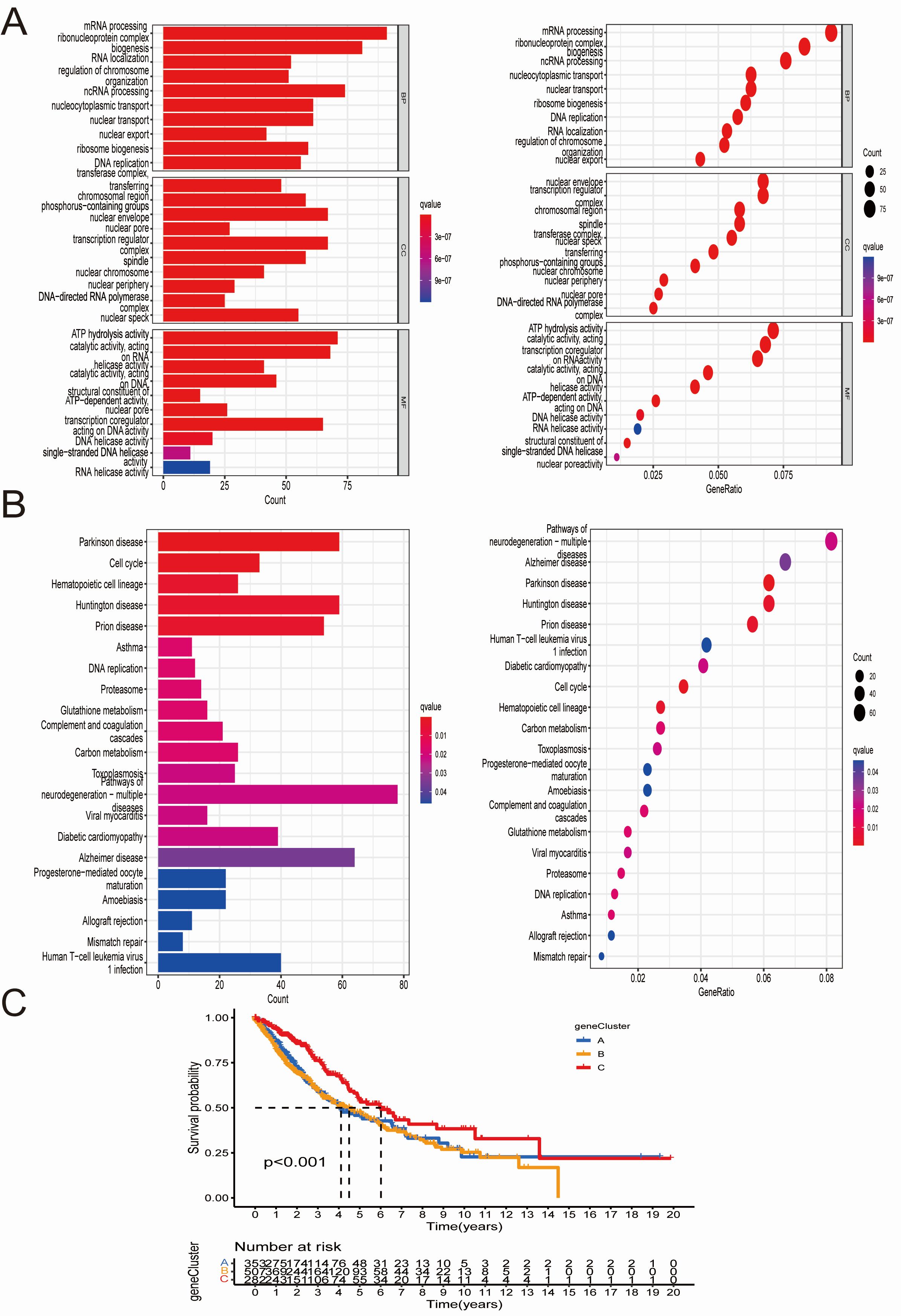

Supplement: Supplementary file 3 — Supplementary Material 3: Figure S3. GSVA analysis performed on CRGs. (A) Inter cluster comparison in terms of distinction in function enrichment pathway. (B) Slide image of lymphocyte infiltration of three clusters. CRGs, cuproptosis related genes; GSVA, Gene set variation analysis [file 12935_2024_3267_MOESM3_ESM.jpg]

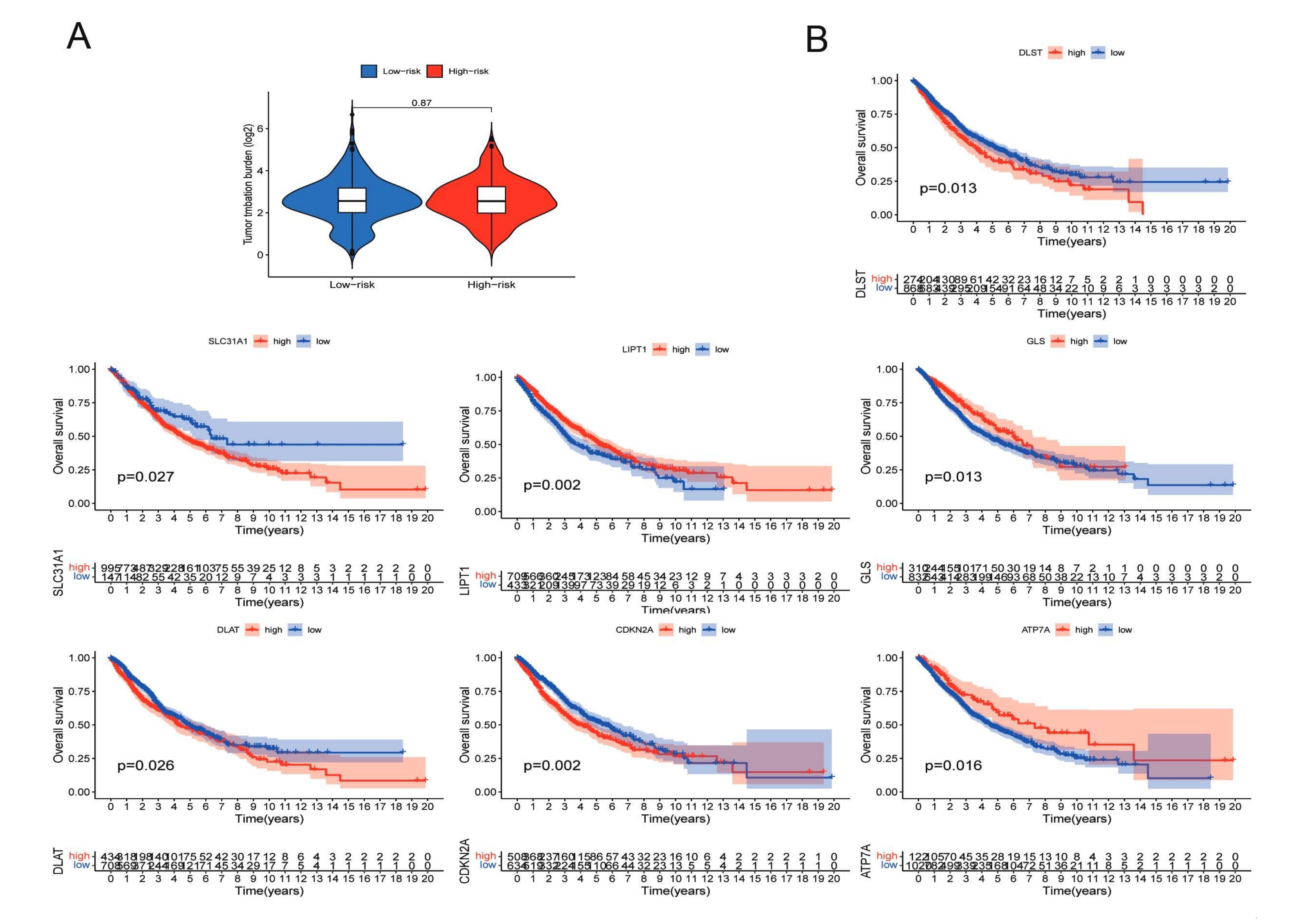

Supplement: Supplementary file 4 — Supplementary Material 4: Figure S4. Acquisition and functional enrichment of CRDEGs. (A) GO functional enrichment of CRDEGs. (B) KEGG functional enrichment of CRDEGs. (C) Survival curve of three gene clusters. (*p < 0.05; **p < 0.01; ***p < 0.001; Ns, not significant). CRDEGs, cuproptosis related differentially expressed genes; GO, gene ontology; KEGG, Kyoto Encyclopedia of Genes and Genomes [file 12935_2024_3267_MOESM4_ESM.jpg]

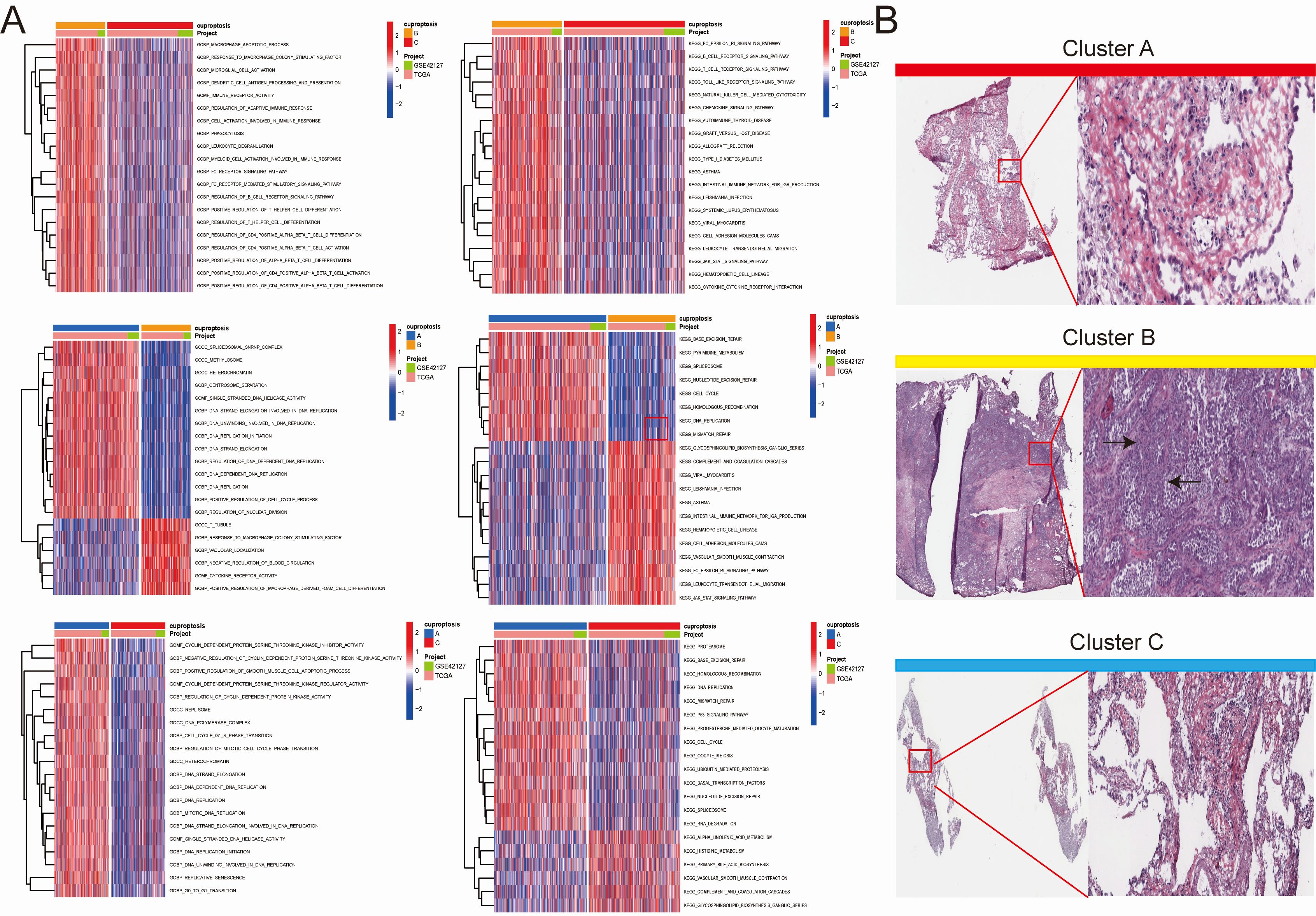

Supplement: Supplementary file 5 — Supplementary Material 5: Figure S5. (A) Drug sensitivity of the high/low-groups of the prognostic score model. [file 12935_2024_3267_MOESM5_ESM.jpg]
