# Supplementary material for: A cuproptosis score model and prognostic score model can evaluate clinical characteristics and immune microenvironment in NSCLC
Source: Cancer Cell Int. 2024 Feb 10;24:68. doi: 10.1186/s12935-024-03267-8 (PMC10859031; doi:10.1186/s12935-024-03267-8)
Supplement: Supplementary file 6 — Supplementary Material 6: Primers [file 12935_2024_3267_MOESM6_ESM.docx]

### Primers

1.

NLRP3-F: 5’-CGTCGTCTTTGAGCCTTCTTG-3’

NLRP3-R: 5’-GATGCTGTCATTGTCCTGGTGT-3’

2.

ATP7B-F: 5’-CTCCGTGTTGGTTGCCTTGA-3’

ATP7B-R: 5’-CCATTTGTCCTCGTGAGTTTGG-3’

3.

ATP7A-F: 5’-AACATTGAACGGAATTTAAGGC-3’

ATP7A-R: 5’-GCTCCAAATCCAAGTTCTCG-3’

4.

SLC31A1-F: 5’-CTCAGGGCAGAGGAAACAAA-3’

SLC31A1-R: 5’-CCTCAATCCAGTAGGCTCATAAC-3’

5.

FDX1-F: 5’-CAAGACAAGGAATGCCATCAG-3’

FDX1-R: 5’-GAAGTTGCAGTGAGCCGAGA-3’

6.

LIAS -F: 5’-CACGAGAATTACTTGAGCCTGTG-3’

LIAS -R: 5’-CAGCATTATGGCCTTGGTGTT-3’

7.

LIPT1-F: 5’-TAGTACTGATGGGACGTTCTTGTC-3’

LIPT1-R: 5’-CATCCGTTGGGTTTATTAGGTG-3’

8.

LIPT2-F: 5’-CATCCGTTGGGTTTATTAGGTG-3’

LIPT2-R: 5’-ATTGTGGCTTCCAGCTTGTCT-3’

9.

DLD -F: 5’-CAGTGCCTCTGAGAACTTACGC-3’

DLD -R: 5’-GTACTCTTCTGCTCCATCATCTTGT-3’

10.

DLAT -F: 5’-GCGACGGGCTCAGAATGTAG-3’

DLAT -R: 5’-AGTCACGCTGTTGCGACGAG-3’

11.

PDHA1-F: 5’- GCCGAATGGAGTTGAAAGCA-3’

PDHA1-R: 5’-GGGGTGAAAGTAAAGCCGTGAG-3’

12.

PDHB -F: 5’-TGATAAATATGCGTACCATTAGACC-3’

PDHB -R: 5’-GCACCAGTGACACGAACAGC-3’

13.

MTF1-F: 5’-TTATTCACATCCCAGGAGCATC-3’

MTF1-R: 5’-AGGAAGGAGCAACACCCAAC-3’

14.

GLS -F: 5’-CTGAGCCCTGAAGCAGTTCG-3’

GLS -R: 5’-GAGACCAGCACATCATACCCATAA-3’

15.

CDKN2A-F: 5’-TGGACACGCTGGTGGTGCT-3’

CDKN2A -R: 5’-TCTAAGTTTCCCGAGGTTTTCTCA-3’

16.

DBT -F: 5’-GGTATATGGGTTGAAGTAATGTAGGC-3’

DBT -R: 5’-GTGCTAGGGTGTAGGGAGGG-3’

17.

GCSH -F: 5’-TGGAACAGTGGGAATCAGCA-3’

GCSH -R: 5’-GATCAGCCAACCATCTTCATAAC-3’

18.

DLST -F: 5’-CTCCTTATGCCAGGGACCAG-3’

DLST -R: 5’-AAGAGCTTCAATCACGCCATTT-3’
